# Prescribed RNA Particle Vaccine Against Porcine Sapovirus Enhances Virus-Neutralizing Antibody Titers in Colostrum and Milk

**DOI:** 10.3390/pathogens15060564

**Published:** 2026-05-23

**Authors:** Bikash Aryal, Sarah Snelson, Thomas Petznick, Qiuhong Wang

**Affiliations:** 1 Center for Food Animal Health, Department of Animal Sciences, College of Food, Agricultural and Environmental Sciences, The Ohio State University, Wooster, OH 44691, USA; aryal.26@osu.edu (B.A.); kinder.91@osu.edu (S.S.); 2 Veterinary Health Services, Omaha, NE 68132, USA; 3 Department of Veterinary Preventive Medicine, College of Veterinary Medicine, The Ohio State University, Columbus, OH 43210, USA

**Keywords:** porcine sapovirus, vaccination, viral neutralization antibody

## Abstract

Confirmed porcine sapovirus (PoSaV) infection in young pigs have been increasing in swine farms; however, studies evaluating strategies to control PoSaV under field conditions remain scarce. In a commercial farm, one group of pregnant sows and gilts (*n* = 13 sows and 2 gilts) were vaccinated intramuscularly twice with an RNA particle vaccine containing the PoSaV VP1 gene, while the other group served as non-vaccinated controls. We evaluated the viral neutralizing (VN) antibodies in colostrum and milk collected at 1, 2, and 3 weeks post-farrowing. Fecal PoSaV shedding in 2-week- and 3-week-old piglets from both groups was assessed by reverse transcription-quantitative PCR. Body weight was measured at birth and 3 weeks of age when piglets were weaned. The vaccination group had higher VN antibody titers than the non-vaccinated group in colostrum and milk samples, with significance for colostrum and milk samples at 1-week post-farrowing. The colostrum and milk samples from both groups contained high VN antibody titers (>10^3.5^ VNT_50_/mL) and remained high for at least three weeks, suggesting previous exposure to PoSaV. Corresponding to these high VN titers, all piglets were negative for PoSaV and had similar body weights at birth and 3 weeks of age. Future vaccination-challenge studies are warranted to confirm the efficacy of this vaccine.

## 1. Introduction

Porcine sapovirus (PoSaV) causes gastroenteritis problems in younger piglets, particularly affecting the weaning and post-weaning piglets. This virus belongs to *Sapovirus* genus within the *Caliciviridae* family and 8 out of 19 genogroups of SaVs are reported to infect various swine populations across the globe [1]. Among these, genogroup III (GIII) is the most prevalent genogroup at swine farms in the United States [2,3]. The Cowden strain, prototype of the GIII sapovirus, has been successfully propagated in various cell lines, including LLC-PK1 and ST cells [2,4]. Although first detected in a 27-day-old diarrheic piglet from an Ohio swine farm decades ago [5], PoSaV is historically a neglected enteric pathogen in swine. However, the confirmed cases of PoSaV infections are increasing in recent years in commercial swine farms, causing gravy or toothpaste like diarrhea in young piglets [3]. In a herd with ongoing piglet diarrhea, 82.6% of litters tested RT-qPCR-positive for PoSaV, with litters showing diarrhea having low CT values (average 15.9), indicating substantially higher viral loads in the affected litters [6]. At the recent “Porcine Sapovirus Symposium” organized by the Swine Health Information Center (SHIC), Dr. Will Fombelle calculated the economic cost of PoSaV infection at farms: piglets suffering from SaV infection were about 1 lb (454 g) less in body weight (BW) when they weaned. So, about $22.5/sow/year of economic losses ($0.75/pig × 12 pigs/litter = $9/litter; $9 × 2.5 litters/year/sow = $22.5/sow/year) could occur at a farm suffering from PoSaV endemics [7]. Despite its economic importance, this pathogen has received limited attention, thereby limiting research efforts and preventive strategies against this virus.

Although this virus predominantly causes diarrhea in weaning and post-weaning pigs [3], only two customized RNA particle vaccines are currently available—from Merck Animal Health (Rahway, NJ, USA) and Medgene (Brookings, SD, USA)—and veterinarians use them to induce lactogenic immunity in sows and gilts, aiming to prevent PoSaV-related diarrhea on farms. Considering the cost of the prescribed Merck PoSaV RNA particle vaccine ($1.32 per dose × 2 doses = $2.64 per gilt/sow), it may bring benefits for swine farmers. However, the immune responses in sows/gilts and efficacy of this vaccine have not been evaluated. The objectives of this study were to evaluate viral neutralizing (VN) antibody responses in the colostrum/milk of gilts/sows immunized with the PoSaV vaccine and correlate the VN antibody titers with piglet performance, including PoSaV shedding and BW.

## 2. Materials and Methods

### 2.1. Vaccination and Sample Collection

A commercial farm with standard cleaning, sanitation, and biosecurity practices, but having a prior history of PoSaV infection, decided to vaccinate some pregnant sows/gilts to fight PoSaV diarrhea. In late December 2022, weaning pigs on the farm suffered from diarrhea and PoSaV BA43 (GenBank accession no. PZ294618) was detected from a 16-day-old diarrheic pig fecal sample collected on December 26 by RT-qPCR by the Veterinary Diagnostic Laboratory at Iowa State University, but not porcine enteric coronaviruses (PEDV, PDCoV, and TGEV) or rotavirus groups A, B and C. However, no subsequent samples have been submitted for sapovirus diagnostic testing since that time. At the time of vaccination (end of March 2025), the farm had approximately 650 sows and gilts with a 4-week batch farrowing system. For vaccination, 26 sows with various parity numbers (1–7) and four gilts were randomly selected and divided into two groups [vaccination, *n* = 15 (13 sows and 2 gilts); non-vaccination, *n* = 15 (13 sows and 2 gilts)]. An prescribed RNA particle PoSaV vaccine that utilized an alphavirus replicon vector and the VP1 gene of PoSaV to express the major viral capsid protein VP1 in the host to induce targeted immune responses against PoSaV was ordered via the Sequivity prescription platform from Merck (https://www.merck-animal-health-usa.com/hub/sequivity/; last accessed on 9 May 2026) and used for vaccination. A “prescribed vaccine” refers to a custom veterinary biological product manufactured on-demand under a written prescription from a licensed veterinarian, as defined by USDA Center for Veterinary Biologics (CVB) in Memorandum 800.214 on Prescription Platform Product Biologics [8]. Unlike conventional off-the-shelf vaccines with fixed, standardized formulations sold broadly to multiple users, a prescribed vaccine is customized for a specific herd, farm, or animal population based on the veterinarian’s assessment of that particular farm’s needs.

**Figure 1:**
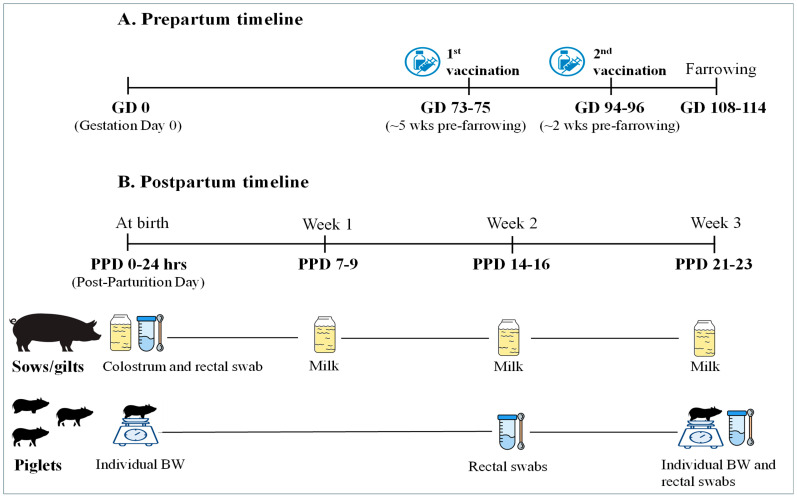
Schematic diagram of the vaccine study in pregnant gilts and their newborn suckling piglets.

Confirmation from Merck Animal Health indicated that the VP1 sequence of the prescribed vaccine shared 98.16% of sequence identity with the PoSaV strain BA43 detected from that farm. For the sows/gilts in the vaccination group, the vaccine was intramuscularly injected twice, three weeks apart, at gestation day (GD) 73–75 (about 5 weeks pre-farrowing) and at GD 94–96 (about 2 weeks pre-farrowing). The sows/gilts in the non-vaccination group were not injected with anything. The BWs of individual piglets in both groups were obtained at birth and at 3 weeks of age, when the piglets were weaned (Figure 1). Colostrum and milk samples on parturition day and 1 week, 2 weeks, and 3 weeks post-farrowing were collected from sows/gilts for VN antibody titers by a TCID_50_-reduction assay. Rectal swab samples from all sows/gilts on parturition day and from 3 piglets per sow at 2 weeks and 3 weeks of age were collected to test PoSaV shedding.

### 2.2. Colostrum and Milk Sample Processing for Whey

The colostrum and milk samples were treated with 2.5 µg/mL of the fungal Rennet from Mucor miehei (Sigma-Aldrich, St. Louis, MO, USA) at 37 °C for 30 min, followed by centrifugation at 2000× *g* for 15 min as described previously [9]. The whey, which was the clear fluid between the curd and fat layer, was taken and heat-inactivated at 56 °C for 30 min prior to testing for VN antibodies by viral neutralization assay.

### 2.3. Virus Neutralization Assay

The VN antibodies to GIII PoSaV in the whey samples were titrated using TCID_50_-reduction virus neutralization assay, as described previously [10]. Briefly, serially diluted whey/serum samples were mixed with 100 TCID_50_ (confirmed by back-titration) of PoSaV Cowden strain per well for 90 min at room temperature before inoculating the whey/serum–virus mixture to the LLC-PK1 cell monolayers in 96-well plates, 4 wells/dilution. The growth medium (GM) for LLC-PK1 consisted of advanced minimum essential medium (AMEM) (Gibco, Carlsbad, CA, USA), 1% antibiotic–antimycotic (Gibco, Carlsbad, CA, USA), 1% L-glutamine (Gibco, Carlsbad, CA, USA), and 5% fetal bovine serum (FBS, HyClone, Logan, UT, USA). Fully confluent cells in 96-well plates were first washed and incubated with the maintenance medium (MM) (GM minus FBS), and incubated for 1 h at 37 °C. Subsequently, the MM was removed, and the cells were inoculated with several whey dilution–virus mixture (50 µL/well) for 60 min. After that, 50 µL of MM containing 200 μM of glycochenodeoxycholic acid (GCDCA) (Sigma-Aldrich, St. Louis, MO, USA) [11] was added to each well, resulting in a final GCDCA concentration of 100 μM. The serum samples from pigs inoculated with the culture medium and PoSaV Cowden strain [12] were used as negative and positive controls, respectively. The cells were then incubated at 37 °C for four days and immunostained for PoSaV-infected cells at 4 days post-inoculation (dpi) by histochemical staining. Briefly, the cells were first fixed with 3.7% formalin for 30 min and permeabilized with 1% Triton X-100 for 5 min. Subsequently, the cells were washed three times with phosphate-buffered saline (PBS) and blocked with 2% non-fat dry milk (NFDM) for 1 h at room temperature to prevent nonspecific binding. Cells were then incubated with primary antibody, guinea pig hyperimmune serum against the virus-like particles of PoSaV Cowden strain (a gift from Dr. Linda J. Saif), for 1 h at 37 °C. After washing the unbound primary antibody with 0.05% PBS-Tween 20 (PBST) for five times, the secondary antibody, horseradish peroxidase (HRP)-conjugated goat anti-guinea pig IgG (H+L) (Invitrogen, Frederick, MD, USA), was added and incubated at 37 °C for 1 h. Cells were again washed five times and stained with a substrate aminoethylcarbazole (AEC; AEC staining kit, Sigma-Aldrich, St. Louis, MO, USA), as described previously [13]. The VN antibody titers were expressed as the reciprocal of the highest whey sample dilution in which there was no virus replication in 50% of the wells (4 replicates) in VNT_50_/mL, as calculated by the Reed–Muench method [14]. 

### 2.4. RNA Extraction and Reverse Transcription (RT)-TaqMan Real-Time PCR (RT-qPCR)

Rectal swabs collected from sows/gilts and piglets were diluted in 1 mL of diluent, and RNA was extracted using MagMaxTM—96 Viral Isolation Kit (Applied Biosystems, Foster, CA, USA) according to the manufacturer’s instructions with minor adjustments, as described previously [2]. Plates were processed on the MagMax™ Express machine (Applied Biosystems, Bedford, MA, USA) (up to 2 plates/run) for automated RNA extraction, involving binding, washing, and elution steps completed in approximately 25 min. Briefly, samples were lysed in a lysis/binding solution concentrate mixed with carrier RNA and 100% isopropanol, followed by the addition of magnetic RNA Binding Beads with lysis/binding enhancer. Subsequent wash steps were carried out using wash solution 1 and wash solution 2, and RNA was eluted in elution buffer for downstream applications.

An RT-qPCR assay was developed utilizing previously published GIII PoSaV-specific primers and probe, including the forward primer (PSapV-F: 5′-AACGCRGTGGCAACGTACAA-3′), reverse primer (PSapV-R: 5′-GCCTCCATCACGAACACTTC-3′), and probe (PSapV-P: 5′-FAM-TGGCTCYTCATCTTCATTGGTGGGAGC-TAMSp-3′) [15], targeting the RdRp–VP1 junction region, using QIAGEN One-Step RT-PCR Kit and RealPlex real-time thermocyclers (Eppendorf, Barkhausenweg, Hamburg, Germany). Each 20 μL reaction mixture consisted of 2 μL of extracted viral RNA, 4 μL of 5× PCR buffer, 0.8 μL of 10 mM dNTP mix, 0.4 μL of each primer (20 μM), 0.4 μL of probe (10 μM), 0.8 μL of RT-PCR enzyme mix, 0.2 μL of RNase inhibitor (40 U/μL), and 11 μL of RNase-free water. Reverse transcription was carried out at 50 °C for 30 min, followed by an initial denaturation step at 95 °C for 15 min. Amplification was then conducted over 40 cycles consisting of denaturation at 95 °C for 15 s, annealing at 55 °C for 20 s, and extension at 60 °C for 45 s.

### 2.5. Statistical Analysis

The VN titers and BW between the vaccination and non-vaccination groups were compared using Student’s *t*-test in GraphPad Prism, version 10.0. Significance levels were set at a *p* < 0.05.

## 3. Results

### 3.1. PoSaV-Specific VN Titers

**Figure 2:**
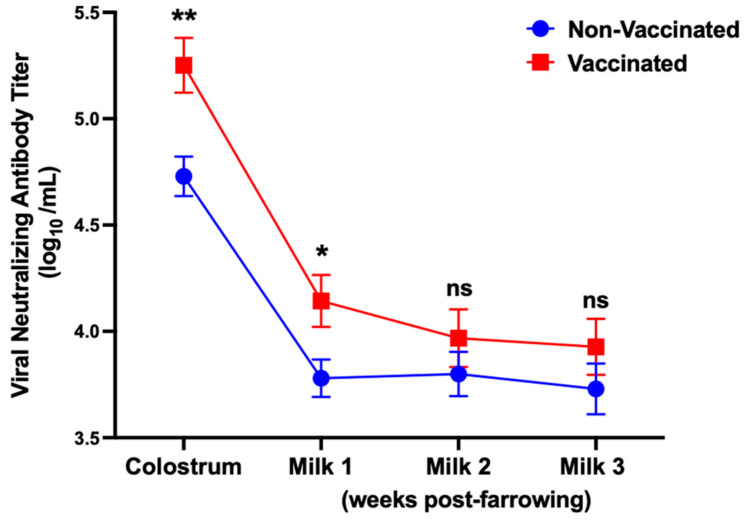
Viral neutralizing antibody titers in the colostrum and milk samples collected at 1 week, 2 weeks, and 3 weeks post-farrowing. The red and blue lines represent the vaccinated sows/gilts and non-vaccinated sows/gilts, respectively. Data are presented as mean ± standard error of mean (SEM). ns: Not significant, *p* > 0.05; * *p* < 0.05; ** *p* < 0.01.

The results showed that both sows and gilts (*n* = 15), regardless of parity, had similar VN antibody titers at different time points within the group. Therefore, the data from sows and gilts were combined for analysis. In every sampling time point, the vaccination group had higher VN antibody titers than the non-vaccinated group, and the differences were significant for colostrum and milk samples at 1-week post-farrowing (Figure 2). The colostrum and milk samples from both groups contained high VN antibody titers (>10^3.5^ VNT_50_/mL), and the titers in both groups remained high for at least three weeks.

### 3.2. Viral Shedding and Body Weight

**Figure 3:**
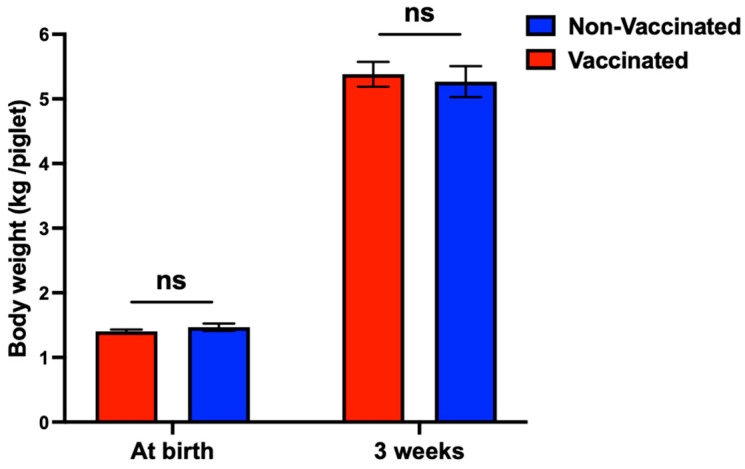
Piglet body weights were compared between vaccinated and non-vaccinated groups at birth and at 3 weeks of age using multiple unpaired *t*-tests. Data are presented as mean ± standard error of mean (SEM). ns: Not significant (*p* > 0.05).

All rectal swab samples of sows/gilts at farrowing and piglets at 2 weeks and 3 weeks post-farrowing were tested negative for PoSaV by RT-qPCR. Piglets from both the vaccinated and non-vaccinated sows/gilts had similar body weights at birth (1.40 vs. 1.47 kg) and at 3 weeks of age (5.38 vs. 5.27 kg) (Figure 3). These results suggest that no PoSaV was circulating within this batch of litters during this study period.

## 4. Discussion

Vaccination of commercial swine populations against PoSaV is not commonly practiced, likely due to underdiagnosis, limited perceived economic impact, and limited vaccine availability. A previous field study in an endemically infected herd demonstrated that maternal vaccination of pregnant sows and gilts with a herd-specific RNA particle PoSaV vaccine reduced fecal viral RNA shedding in both sow and gilt litters and pre-weaning mortality in piglets in gilt but not sow litters; however, vaccine-induced virus neutralization antibody responses were not evaluated [16]. Moreover, in a separate study, the immunization of sows with PoSaV virus-like particles (VLPs) induced high serum antibody titers after booster vaccination, and significantly reduced viral shedding in their piglets following oral challenge with the PoSaV CH430 strain, demonstrating passive protection [17]. Although this experimental study on the VLP vaccine demonstrates protection in piglets, there is limited information from field studies evaluating the efficacy of prescribed commercial PoSaV vaccines on lactogenic immunity. This study assessed the protective efficacy of a prescribed RNA particle vaccine in sows and gilts on a commercial swine farm, based on VN antibody responses in colostrum and milk, viral shedding, and piglet growth performance.

Neonatal piglets against infectious pathogens rely on maternal immunity via colostrum initially but depend on mature milk thereafter. More specifically, for PoSaV, the peak disease occurs at the weaning to post-weaning stage of piglets, since PoSaV is universally distributed on swine farms [3] and sows/gilts can provide lactogenic immunity during the nursing stage. This was confirmed again in this study. We studied the kinetics of VN antibody titers in colostrum and milk samples collected at 1 week, 2 weeks, and 3 weeks post-farrowing to evaluate the duration of protection. In our vaccine trial, both vaccinated and non-vaccinated sows/gilts exhibited high titers of PoSaV-specific VN antibodies in the colostrum and milk samples, indicating prior natural exposure to PoSaV infection before vaccination. Correspondingly, all piglets were tested and shown to be free of PoSaV infection, suggesting the absence of active viral circulation during the study period. Alternatively, the high lactogenic VN antibody titers present in both groups during the nursing period may themselves have been sufficient to suppress active viral replication and shedding in piglets.

The exact timing and duration of the most recent natural exposure to PoSaV in that farm remain unknown. PoSaV was last detected on the farm by RT-qPCR in the end of December 2022, and no diagnostic testing was conducted between that time and the initiation of this study at the end of March 2025. The persistence of high VN antibody titers in non-vaccinated sows/gilts at the time of farrowing suggests that additional undocumented infections of PoSaV may have occurred after December 2022, although we do not know when these animals were infected due to the lack of interim diagnostic surveillance. Moreover, how long maternally derived neutralizing antibodies, following natural infection with PoSaV in sows or gilts, can last and be transferred to piglets across successive parities remains unclear and warrants further investigation. Previous studies on other swine enteric viruses such as transmissible gastroenteritis virus (TGEV) and porcine epidemic diarrhea virus (PEDV) indicate that lactogenic immunity declines over time, and its persistence across successive parities is not well defined [18].

The administration of the Merck PoSaV vaccine further boosted pre-existing PoSaV-specific immune responses in vaccinated sows/gilts that had significantly higher (~1 log_10_-higher) VN antibody titers in colostrum and milk at 1 week post-farrowing than the non-vaccinated sows/gilts. This observation is biologically plausible given that the vaccine VP1 shares 98.16% of sequence identity with the farm outbreak strain BA43, resulting in the enhanced passive protection of piglets during the first week of life. In addition, the high VN antibody levels observed in both vaccinated and non-vaccinated groups likely conferred passive lactogenic immunity sufficient to protect piglets from PoSaV infection under farm conditions. However, because sows/gilts and piglets from both groups did not shed PoSaV, the protective efficacy of the vaccine could not be definitively determined in this field setting. Diarrhea caused by PoSaV in piglets is estimated to reduce weaning body weight by 0.66 kg per piglet [6]. The comparable body weight observed between piglets from vaccinated and non-vaccinated sows/gilts at birth and at 3 weeks of age corresponds to the absence of PoSaV infection at those time points, as confirmed by the RT-qPCR results on rectal swabs.

Several limitations of this field study should be acknowledged. First, pig serum samples were not available, including the serum samples from sows and gilts prior to vaccination, preventing direct assessment of vaccination-induced antibody increases relative to baseline titers. Second, both groups of animals had high VN antibody titers at all time points (>10^3.5^ VNT_50_/mL), indicating prior natural exposure to PoSaV; the absence of seronegative naive animals prevented the evaluation of vaccination-induced primary immune responses in PoSaV-unexposed animals. Because PoSaV infections in adult animals are often subclinical, the high baseline neutralizing antibody levels observed in both vaccinated and non-vaccinated sows/gilts were not unexpected under endemic farm conditions. Third, the exact timing and extent of prior natural PoSaV exposure on this farm remain unknown, as no diagnostic testing was conducted between December 2022 and the initiation of this study in March 2025, limiting interpretation of the vaccine efficacy. Fourth, the absence of active PoSaV circulation during the study period, as confirmed by RT-qPCR negativity in all rectal swab samples, prevented definitive assessment of vaccine-mediated protection against PoSaV infection and disease in piglets. The lack of detectable infection in nursing piglets during this period may have also been influenced by the high levels of maternal neutralizing antibodies present in colostrum and milk. Fifth, the observation and sample collection period was limited to 3 weeks post-farrowing due to the farm’s weaning schedule; collecting additional rectal swab samples from weaned pigs at 4 and 5 weeks of age may provide additional information on vaccine efficacy, since these age groups are more susceptible to PoSaV infection and diarrhea than nursing piglets due to decreasing maternal antibody levels and weaning-associated stress. Sixth, whether weaning pigs are another vaccination target besides pregnant sows and gilts to reduce PoSaV-caused economic losses on swine farms remains to be evaluated. Finally, as this study was conducted on a single commercial farm under endemic conditions, the findings may not be directly generalizable to herds with different PoSaV exposure histories or immune status. Nevertheless, the swine industry would like to know whether this vaccine is effective and cost-beneficial. Therefore, it is both novel and significant to evaluate neutralizing antibody levels in vaccinated and non-vaccinated sows/gilts, particularly given the limited funding available for this neglected virus.

In summary, vaccinating pregnant sows/gilts against PoSaV boosted the lactogenic immunity and may protect neonatal piglets from PoSaV infections. Future controlled vaccination-challenge studies incorporating seronegative sows/gilts, pre-vaccination serum collection, and post-weaning follow-up are warranted to comprehensively evaluate vaccine efficacy. Moreover, currently available vaccines against PoSaV are RNA particle-based and just target the viral VP1 protein. As virus isolation becomes more efficient [2], it may facilitate the development of whole-virus vaccines, including autogenous vaccines, as future commercial vaccine options.

## Data Availability

The raw data supporting the conclusions of this article will be made available by the authors on request.
